# Grape Pomace as a New Coagulant for Tofu Production: Physicochemical and Sensory Effects

**DOI:** 10.3390/foods10081857

**Published:** 2021-08-11

**Authors:** Giuseppe Zeppa, Martina Tedesco, Marta Bertolino, Betül Çilek Tatar

**Affiliations:** 1 Department of Agriculture, Forestry and Food Sciences (DISAFA), University of Turin, 10095 Grugliasco, Italy; martina.tedesco@unito.it (M.T.); marta.bertolino@unito.it (M.B.); betul.cilek@ankaramedipol.edu.tr (B.Ç.T.); 2 Department of Gastronomy and Culinary Arts, Ankara Medipol University, Ankara 06050, Turkey

**Keywords:** tofu, grape pomace, coagulation, polyphenols, radical scavenging activity, texture, consumer acceptability

## Abstract

Tofu, one of the most important products made from soymilk, is obtained through a coagulation process performed with various coagulants (acids, salts and, enzymes). In this study, innovative tofu samples were produced using the grape pomace (GP) powders of different varieties (Barbera, Chardonnay, Moscato, and Pinot Noir) with different origins (fermented and distilled) at two concentration levels (2.5% and 5% *w*/*v*) as coagulants, and comparisons with traditional tofu were made. Physicochemical characteristics, phenolic contents, radical scavenging activity levels, textural properties, and consumer acceptability were evaluated. The moisture, protein content, and pH levels of GP tofu samples were slightly lower than those of traditional tofu. Regarding textural parameters, except for hardness, all other parameters were significantly lower in GP tofu samples, with differences due to GP concentration. The colours of GP tofu varied from amber-yellow to violet according to the GP origin. The blue-violet colours were observed predominantly in tofu samples obtained with Barbera and Pinot Noir GPs, while the other GP tofu samples showed amber-yellow colours. The concentrations of polyphenols were 2–10 times higher than in traditional tofu, while the radical scavenging activity levels were 9–80 times higher. The GP tofu samples were favoured by consumers, with small differences among the GP varieties.

## 1. Introduction

Tofu, soybean curd, or bean curd is one of the most important soya products and is produced by the addition of coagulants to soymilk. It is widely consumed in Asia and has been growing in popularity worldwide because of its high nutritional value, good texture, and unique flavour [1]. It has high contents of highly digestible proteins, dietary fibre, soy saponins, and isoflavones that can prevent the progression of arteriosclerosis by reducing plasma lipids [2,3,4]. Furthermore, tofu is cholesterol-free and has a lower amount of saturated fat than animal sources of protein, such as meat or milk [5]. 

There are two major steps in tofu production: heating soymilk and coagulation of soymilk to obtain a curd [1,6]. 

Heating soymilk causes denaturation of soy protein and exposure of the inner hydrophobic groups [7]. Electrostatic repulsion extensively opposes protein–protein interactions, preventing gel formation, while the balance of attraction and repulsion forces in protein molecules determines the stability of soymilk [7]. The heating of soymilk is also an essential step in the denaturation of antinutritional compounds [8]. Coagulation is then achieved by the addition of a coagulant, while hydrophobic interactions of the denatured protein lead to the aggregation of protein molecules, thereby forming curd [9,10].

The physical, textural, and sensory properties of the obtained tofu are affected by multiple factors that can be classified as intrinsic (i.e., composition of soya seeds) and extrinsic (i.e., processing conditions and packaging) factors [11,12,13]. The most critical step in the processing is the coagulation phase, which involves the selection of a coagulant [6,14,15,16]. At present, common coagulants are divided into three main groups: acids, salts, and enzymes [6,13]. The driving forces behind the acid gelation of soy proteins are isoelectric precipitation, including salt bridging, and direct interactions, such as hydrogen bonding and van der Waals forces [8,16]. With salts, a three-dimensional network structure is formed by forming salt bridges to cross-connect protein molecules [6]. In particular, phytic acids interact with Ca^2+^ to form non-ionising products that allow interactions between Ca^2+^ and proteins. Then, Ca^2+^ interacts with non-particulate proteins to form new protein particles, which are associated with each other to develop the gel network [17]. Enzyme coagulants such as transglutaminase can assemble proteins with the help of isopeptide bonds that are formed from the amine group in the glutamine residue and the ξ-amino group in the lysine residue [11]. Fermented soy whey, glucose-delta-lactone (GDL), and citric acid are commonly used acidic coagulants [6,14,16,18], while calcium sulphate, calcium chloride, calcium acetate, calcium lactate, and magnesium chloride are commonly used salt coagulants [5,19]. Magnesium chloride allows the taste of soybean to be retained and creates a more natural flavour for tofu; however, it is a quick-acting coagulant with a lower yield, forming a harder and non-uniform tofu [5].

Many studies have been devoted to evaluating the effects of these different coagulants on tofu quality. Shi et al. [6] studied the effects of four different coagulants (magnesium chloride, calcium sulphate, GDL, and fermented soybean whey) on the gelation behaviour of tofu, and their results showed a significant effect on the physicochemical properties, textural characteristics, and volatile flavour profile. In particular, the calcium sulphate tofu had the best overall acceptance in terms of sensory evaluation, while magnesium chloride tofu displayed the best gumminess, resilience, and mouthfeel. 

Zhao et al. [18] evaluated the effects of different salts (potassium chloride, calcium chloride, and calcium sulphate) on the gelation of citric-acid-induced tofu and showed that this addition improved the mechanical properties of the product. In fact, the balance between forces that are suitable for gel structure leads to an increase in the storage modulus.

Cao et al. [16] studied the effects of different organic acids (citric, malic, and tartaric) on tofu quality, highlighting that tofu prepared with organic acids is comparable to that obtained with GDL, although the product prepared with tartaric acid had comparatively poorer physical properties and less robust chemical interactions. Acidification was also obtained with commercial lactic cultures, which in comparison with GDL showed a significantly earlier gel point but a similar final gel structure [8].

The structure and formation mechanism of tofu induced by different types of coagulants (GDL, calcium sulphate (CS), and microbial transglutaminase (MTGase)) were studied by Wang [20]. MTGase produced gels with an intermediate structure between CS and GDL but with the best uniformity. Similar work was performed by Rui et al. [21] who investigated the effects of different coagulants (GDL, magnesium chloride (MC), and MTGase) on the degradation of soybean proteins; their results showed that the MTGase tofu had the lowest protein digestibility when compared to the gels prepared with MC and GDL. Ezeama and Dobson [22] used Epson salt, lime, and tamarind to make tofu, showing that the fresh product obtained with Epson salt was well received by consumers because of its high calcium, magnesium, and protein contents.

Mixing different types of coagulants can potentially overcome the disadvantages of a single coagulant and improve the quality of tofu [1,5,13,18,20,23], although research efforts have also explored new coagulants to replace traditional ones. Crab shell powder [24], roselle water extract [25], trimagnesium citrate [26], papain [27], and commercial rennet have been used [28]. As highlighted by Zheng et al. [13], the use of a new coagulant requires not only the evaluation of its impacts on the compositional, textural, and sensory characteristics of tofu, but also its feasibility according to local availability and production costs; therefore, the purpose of this study was to evaluate the possibility of using grape pomace (GP) as a coagulating agent for tofu production. The GP, composed of a mix of grape seeds and skins, corresponds to approximately 62% of the total waste generated during the winemaking process [29]. GPs are partially distilled for ethanol extraction, although most of this by-product is discarded, with several environmental and economic effects [29]. Nevertheless, GPs are rich sources of high-value compounds, such as acids (tartaric, malic, and citric), fibres, polyphenols, and salts, and have been widely suggested as ingredients in pasta, puree, biscuits, yoghurt, cereal bars, pancakes, and cheese [29]. Moreover, distilled GPs are rich sources of high-value compounds, although in contrast to fresh GPs, no applications have been suggested for distilled GPs; therefore, the problem of the disposal of fresh and distilled GPs still needs to be solved, and their use as ingredients in foods facilitates green production and the minimisation of by-product treatment costs, creating new sources of income for grape producers and increasing consumer interest in healthier foods. Furthermore, the addition of grape by-products to foods may represent a novel strategy to produce functional foods with high contents of polyphenols and high antioxidant activity. Since high concentrations of acids and salts are present in GPs [29], these by-products could act as coagulants for soy proteins. The possibility of their use in tofu production is very good in regard to reducing winemaking waste and obtaining new and innovative functional foods with high contents of polyphenols that are completely produced from vegetables. 

## 2. Materials and Methods

### 2.1. Chemicals

Folin and Ciocalteu’s phenol reagent, sodium carbonate (≥99.5%), 2,2′-diphenyl-1-picrylhydrazyl (95%) (DPPH), 6-hydroxy-2,5,7,8-tetramethylchroman-2-carboxylic acid (97%; Trolox), citric acid (≥99.5%), and anhydrous magnesium chloride (≥98%) were purchased from Sigma-Aldrich (Milan, Italy). Gallic acid and ethanol (≥99.9%) were supplied by Fluka (Milan, Italy). Ultrapure water was prepared using a Milli-Q filter system (Millipore, Milan, Italy).

### 2.2. Grape Skin Powder

GPs evaluated for tofu production were obtained from red (Barbera and Pinot Noir) and white (Chardonnay and Moscato) varieties. Fresh GPs of Barbera, Chardonnay, moscato, and Pinot Noir were produced by Cantine Fratelli Batasiolo winemaker (La Morra, Cuneo, Italy). In addition, distilled GPs of Barbera, Chardonnay, and Moscato were evaluated and produced by Distilleria Mazzetti D’Altavilla (Altavilla M.to, Asti, Italy). All GPs were produced during the 2019 vintage. All GPs produced by Distilleria Mazzetti D’Altavilla were fermented, while for GPs produced by Cantine Fratelli Batasiolo, only Barbera was fermented. For each distilled GP, the product was used before distillation, after continuous distillation, and after discontinuous distillation. GPs were sieved to remove grape seeds and the skins obtained were dried in a Memmert UFE550 oven (Schwabach, Germany) at 54 °C for 48 h, then ground with a Retsch ZM200 grinder (Retsch Gmbh, Haan, Germany) and a SassuoloLab ball mill (Fiorano Modenese, Modena, Italy) in order to obtain a powder finer than 20 µm. The powders obtained were vacuum-packaged in sealed polyethylene bags and stored at 4 °C in the dark until tofu production. 

### 2.3. Tofu Production

Soybeans were purchased from Tibiona Farine (Villanova Mondovì, Cuneo, Italy). Soymilk was prepared according to the method described by Shi et al. [6] with slight modifications. Soybeans were washed and soaked in drinking water (Valmora SpA, Luserna San Giovanni, TO, Italy) at a ratio of 1:3 (*w*/*v*) for 18 h at 4 °C. The hydrated beans were rinsed and ground using a MiniPimer blade (Braun MR550CA, Milan, Italy) with warm (50 °C) drinking water (1:5 *w*/*v*) for 20 s. The mixture was boiled for 15 min to remove the foam produced during the boiling procedure and gently mixed. Finally, raw soymilk was obtained by filtration through a 250 µm sieve to separate the okara (soy pulp). Soymilk (500 mL) was heated to 75 °C and coagulants were added. After 25 min of resting, the curds were transferred into a 5 × 5 × 5 cm mould and the tofu curds were pressed at 10 g/cm^2^ for 30 min. The tofu was stored at 4 °C for 12 h. Two control tofu samples were prepared—the first with magnesium chloride (1.83 g/100 mL of soymilk) [5] and the second with citric acid (0.7 g/100 mL of soymilk) [16]. For each GP, two concentrations (2.5% and 5%) were used as coagulants, since a preliminary test showed that tofu production is possible only with at least 2% of GPs. Before use, all coagulants were mixed (1:1 *w*/*w*) with the same drinking water used for soymilk production in order to obtain a coagulating solution. The pH values of these coagulant solutions were 2.52 for citric acid, 7.52 for magnesium chloride, and between 3.56 and 4.09 for the GPs. Three productions were performed for each coagulant.

### 2.4. Chemical Composition

The dry matter content was determined at 120 °C using a Gibertini Eurotherm electronic moisture balance (Gibertini Elettronica, Novate Milanese, MI, Italy) using 3 g of tofu. Total protein content (conversion factor 6.25) was measured using the Kjeldahl method with a UDK 130A system (Velp Scientifica, Usmate, Italy). The ash content was determined in a muffle furnace according to AOAC 942.05 [30], while the lipid fraction was extracted using a Soxhlet Velp Extraction System SER 148 (Velp Scientifica, Usmate, Italy) for 6 h using n-hexane as a solvent. The carbohydrate value was estimated from the difference. A pH meter (MICROpH 2002; Crison, Carpi, Italy) was used for pH measurements. 

Water activity was determined using 2 g of tofu with an AquaLab PRE water activity meter (Decagon Devices, Pullman, WA, USA).

Colour analyses of tofu were conducted according to Rojo-Poveda et al. [31] with a CM-5 spectrocolourimeter (Konica Minolta, Tokyo, Japan) in transmittance and specular-component-excluded (SCE) modes. The colour space parameters L*, a*, and b* (CIELAB values) were used to measure the colorimetric characteristics, where L* is a coefficient of lightness ranging from 0 (black) to 100 (white), a* indicates the red-green colours (red with +a*; green with −a), and b* represents the yellow-blue colours (yellow when positive b*; blue when negative b*). The ΔE parameter, which represents the difference between two colours and is perceptible by the human eye when ΔE > 2.5 [31], was calculated as ΔE = [(ΔL)^2^ + (Δa)^2^ + (Δb)^2^]^0.5^. All analyses were performed in triplicate. The ΔE was calculated using the mean L*, a*, and b* values calculated for each tofu. 

### 2.5. Textural Analysis

Tofu texture analysis was carried out with a TAXT2i Texture Analyser ^®^ (Stable Micro Systems, Godalming, UK) according to the methods used by Zhao et al. [18] and Shi et al. [6]. Samples were cut into 25 × 25 × 25 mm cubes, and each cube was subjected to texture profile analysis (TPA) with two compression cycles using an SMS P/100 probe with a 25 kg load cell. The samples were deformed by 50%, and there was a wait of 5 s between two compressions. A speed of 1 mm/s and an instrumental trigger equal to 0.05 N were used for the test. The Texture Experts Exceeds version 2.54 software package (Stable Micro Systems, Godalming, UK) was used for data acquisition. Hardness, adhesiveness, cohesiveness, gumminess, chewiness, elasticity, and resilience were measured. Analyses were performed in triplicate.

### 2.6. Liking Test 

For each tofu, a consumer evaluation was performed, as in the study conducted by Rojo-Poveda et al. [31], with 28 consumers (10 men and 18 women) who were familiar with and ate tofu. Tasters were asked to evaluate only the overall liking on a 9-point hedonic scale (1 = extremely dislike; 9 = extremely like) [32]. Water was provided for rinsing of the mouth between the sample tastings. Tests were carried out in an adapted air-conditioned room equipped with white light at approximately 21 °C. Tofu samples (5 g) were stored at 4 °C for 30 min prior to serving, cut into 3 × 4 × 1 cm blocks, and placed blinded on a white, non-odorous plastic plate coded with a random three-digit number. Samples were served in a completely randomised order.

### 2.7. Polyphenol Extraction

Polyphenol extraction was performed according to the method described by Ross et al. [33], with slight modifications. Briefly, tofu (0.5 g) was mixed with 5 mL of ethanol–water solution (70/30, *v*/*v*) and the extraction was performed at 25 °C for 1 h with a VDRL 711 orbital shaker (Asal S.r.l., Milan, Italy) under constant rotatory agitation at 60 rpm. All of the extracts were centrifuged at 4000× *g* for 15 min at 15 °C and the supernatants were collected and filtered through a 0.45 μm nylon membrane filter. Samples were stored in amber vials at 18 °C. All extractions were performed in triplicate.

### 2.8. Total Phenolic Content 

The total phenolic (TPC) content was determined according to the method described by Barbosa-Pereira et al. [34] in 96-well microplates using a BioTek Synergy HT spectrophotometric multidetection microplate reader (BioTek Instruments, Milan, Italy). All measurements were performed in triplicate. A calibration curve of gallic acid (20–100 mg/L) was constructed to quantify the concentration, which was expressed in milligrams of gallic acid equivalents per gram of product (mg GAE/g). 

### 2.9. Antioxidant Capacity

The antioxidant capacity of tofu was assessed using the 2,2′-diphenyl-1-picrylhydrazyl (DPPH•) radical scavenging method described by Barbosa-Pereira et al. [34]. All assays were conducted in triplicate in 96-well microplates with a BioTek Synergy HT spectrophotometric multidetection microplate reader (BioTek Instruments). Antioxidant capacity was calculated as the inhibition percentage (IP) of the DPPH radical as follows:

IP (%) = ((A_0_ − A_30_)/A_0_) × 100

where A_0_ is the absorbance at the initial time point and A_30_ is the absorbance after 30 min.

A standard curve of Trolox was constructed (12.5300 μM) for assessment of radical scavenging activity values, which were expressed as millimoles of Trolox equivalents per gram of product (mmol TE/g).

### 2.10. Statistical Analysis

Physicochemical data were subjected to a one-way analysis of variance (ANOVA) with Duncan’s post hoc test at a 95% confidence level in STATISTICA software for Windows (version 13.3; StatSoft Inc., Tulsa, OK, USA). Values obtained using the consumer acceptance test were analysed using the Kruskal–Wallis test (test H) in STATISTICA software for Windows (version 13.3; StatSoft Inc., Tulsa, OK, USA).

## 3. Results and Discussion

### 3.1. Chemical Composition of Tofu

The compositional parameters of the tofu samples are presented in Table 1. Moisture is an important indicator in the production process of tofu, which has a significant influence on the resulting texture and shelf life. The moisture content of the produced tofu was between 78% and 85%, which is higher than the values reported by Shi et al. [6] for tofu obtained with magnesium chloride (57.7%), calcium sulphate (63.3%), or GDL (76.3%), but similar to the values reported by Kim et al. [35] for tofu obtained with calcium sulphate (83.4%) and by Lee et al. [2] for tofu obtained with magnesium chloride (79.3%). With the use of GPs, there was an increase in the dry extract (2–3%) with respect to the tofu obtained with magnesium chloride (MSG) or citric acid (CIT); in particular, the dry extract was highest when the GPs were used at 5%. The wide range of moisture contents for tofu samples obtained with GPs may be attributable to the different compositions of each GP and their effects on the water-holding capacity and gel network structures. 

In addition, the ash content was higher for tofu produced with GPs at 5% due to the presence of salts in the GPs. The varieties and distillation methods did not affect the ash content of tofu. 

With the use of GPs, there was a decrease in the protein content in the tofu when compared to the values obtained with the reference commercial coagulants (MAG and CIT). The lowest protein values were found in the tofu with Barbera (4.81%) and Moscato (4.56%) pomaces, with reductions of up to 4%. Lee et al. [2] reported a protein content of 10.2% for tofu obtained with magnesium chloride, whereas Shi et al. [6] obtained tofu with a protein content between 7.7% and 18.5% according to the coagulant used. Similar results (8.74–12.74%) were obtained by Darmanjana et al. [36] for different tofu samples produced with nigari.

The application of GPs favoured increases in carbohydrate content, which was about 2% for traditional tofu and up to 15% for tofu obtained with Moscato GPs. In contrast, 3.3% carbohydrate was reported for magnesium chloride tofu [2]. These values were high due to the sugar content of non-fermented white GPs, while the highest values were reported when 5% of GPs were used. Fat contents of 0.97%, 3.95%, and higher were obtained with 2.5% GPs. Similar results were obtained by Lee (1.17%) [2]. The lowest quantity of fats was measured for tofu obtained using distilled GPs, as the distillation process can remove fats from grape stones.

The pH values for tofu were between 4.12 (for the product obtained with BA_PD 5%) and 5.47 (for PN 2.5%). These differences were due to the GP composition, since the Barbera (BA) solution used for coagulation showed a pH of 3.57, while the Pinot Noir (PN) solution showed a pH of 4.09. Generally, for the same GP, the pH value of tofu produced with 2.5% GP was higher than that produced with 5% GP. The mean pH values for tofu obtained with magnesium chloride and citric acid were 5.98 and 5.18, respectively, depending on the coagulant type. A value of 5.9 was also reported by Shi [6] for magnesium chloride tofu.

The water activity levels were similar for all products and higher than 1 in all cases.

The results of the colour analysis are reported in Table 2. Having a high L* value is a favourable characteristic, as consumers prefer lighter or whiter tofu [37]; a good coagulant should produce a tofu with a high L* value [14]. Using GPs as coagulants, the colours of obtained tofu samples were significantly different from standard products, characterised by colours that varied from amber yellow to violet. The blue-violet colours were seen above all in Barbera GP tofu, while the other GPs produced amber-yellow colours. Generally, the products obtained with 5% GPs were darker, with higher red (a*) components for the red varieties Barbera and Pinot Noir and higher yellow components (b*) for the white varieties Moscato and Chardonnay. Different colours were produced with the GPs from Barbera. In addition, after distillation, the differences were significant, although this process did not determine significant differences among tofu samples. 

Colour differences among tofu samples were generally perceptible by eye, since the ΔE values were higher than 2.5 for all the samples (Table 3). Lower values of ΔE were obtained for the comparison between the tofu samples produced with white GPs (Chardonnay and Moscato) or for products made with the red grape Barbera.

### 3.2. Texture Analysis of Tofu

The textural properties of tofu are related to the processing conditions, the concentrations of protein and soluble solids in the soymilk, and the type of coagulant used, and they play a critical role in the consumer acceptability of tofu [37]. To understand the textural properties of tofu made with the above-described coagulants, the TPA results, including hardness, adhesiveness, springiness, resilience, cohesiveness, gumminess, and chewiness results, are presented in Table 4.

The texture of tofu obtained with the GPs was significantly different from that of standard products. This effect of coagulant on tofu texture has also been reported by other authors [6,18]. The tofu obtained with GPs showed higher hardness and adhesiveness, but lower cohesiveness, chewiness, resilience, and Friedman cohesiveness.

The hardness value is defined as the maximum force in the first compression cycle or the force required to achieve deformation of a given material, which is a measure of the resistance to the destructive force during compression.

In particular, tofu samples produced with GPs was characterised by hardness values of between 2.40 and 6.30 N, up to six times the upper values of control tofu samples obtained with magnesium chloride or citric acid. Hardness values of 144–339 g/cm^2^ for citric tofu samples with different salts were reported by Zhao [18], while values between 825 and 4334 g/cm^2^ for tofu samples obtained with different coagulants were reported by Shi [6]. While there was no variety effect, when the GP concentration was increased, the hardness also increased, with tofu formulated samples with PN_5% and MO_PD 5% showing the highest values.

In addition, adhesiveness (the work necessary to overcome the attractive forces between the surface of the product and the surface of the other material with which the product comes in contact) was higher for tofu obtained with GPs, showing values of between −0.89 and −3.11 J. The products obtained with 5% GP showed the highest values. No effect was highlighted for the grape variety, although there was an effect due to GP concentration. 

Cohesiveness (the strength of the internal bonds making up the body of the product) was significantly lower for GP tofu compared with the tofu standard. This is an indication of a weakening of the internal bonds of the product and a lower resistance to second compression.

While gumminess levels were similar between GPs and standard tofu, springiness and elasticity were significantly lower for GP tofu samples, with values of between 2.34 and 4.23. In addition, gumminess (the energy needed to disintegrate a semisolid food until it is ready for swallowing) and chewiness (the energy needed to chew a solid food until it is ready for swallowing) were significantly lower for GP tofu samples compared to standard products.

The above results confirm that the coagulant can influence the internal structure of the tofu matrix and the textural characteristics of the product. 

When using GPs, there is acid coagulation due to the high quantities of acids (tartaric, malic, and citric) present in this material [29,38], which is also highlighted by pH values measured on coagulant solutions used for tofu production, although other components, such as potassium and fibre [29,38], could also have significant effects on gel network production. Tofu is a precipitated soy protein that starts coagulating at approximately pH 6.00; therefore, the pH of tofu can be related to its textural properties. It has been reported that pH strongly affects the extent of Ca^2+^ binding because hydrogen ions compete with calcium ions for the same binding sites on the protein molecule. When the rate of acidification is much higher, all the subunits will be incorporated much faster, as their lower isoelectric points will be reached faster. It has been hypothesised that because of the range of isoelectric points of soy protein subunits, at the pH level where some subunits become destabilised and start to aggregate, other subunits still exhibit repulsive charges, hindering the ability of destabilising proteins to form a network. When there is rapid acidification, a gel network will appear only after more protein species have reached their isoelectric point; therefore, the greater the net charge on the protein molecule, the greater the electrostatic repulsion between molecules, preventing the interactions required to form a gel matrix [7,8]. 

GPs are also rich in salts and potassium. The addition of salts increased protein–protein and protein–solvent interactions, leading to the formation of a denser network. Zhang [39] reported that divalent calcium ion–protein bridges contributed to a more compact gel structure and higher gel hardness, although Lu [40] reported that the gel strength increased with the addition of CaCl_2_ while it decreased under excessive Ca^2+^. Monovalent chloride ions (Li^+^, K^+^, Rb^+^, and Cs^+^) form a fine stranded matrix at ionic strengths of less than 0.1 mol, while the salt concentration required to change gel microstructure depends on the salt’s position in the Hofmeister series [41]. The potassium concentration in soymilk due to GP addition is lower than 0.1 mol, meaning that it contributes little to gel network formation, and consequently a reduction in gelation is seen, as highlighted by Zhao [18], when potassium is added to soymilk at low concentrations.

The presence of GP fibre, which can reduce the homogeneity of the gel microstructure, altering the network structure, is also critical for the gelation and final structure of tofu.

### 3.3. Polyphenolic Content and Radical Scavenging Activity

The polyphenolic content (TPC) and radical scavenging activity (RSA) values for the GPs used as coagulants are reported in Table 5. The GPs of the red grape varieties Barbera and Pinot Noir showed the highest values in the studies conducted by Qing et al. [42] and Najak et al. [43].

In addition, the GPs obtained from Barbera and Pinot Noir showed the highest values in our study. During the distillation process, there was a reduction in polyphenol content for Barbera. The GPs obtained using the continuous distillation system showed an RSA higher than that obtained using the discontinuous system. In contrast, the TPC was not influenced by the distillation method. 

When GPs were used as coagulants, there were significant increases in the TPC and RSA values of the obtained tofu samples compared with the standard product (Table 6). The lack of previous data made it difficult to compare our results with other experiments in order to establish the effects of the use of these coagulants on TPC and RSA. The highest values were obtained with 5% GPs, although the obtained values were not twice the values obtained with 2.5% GPs for the leak during the pressing phase.

The obtained values of TPC and RSA, despite these leaks in the pressing phase, were very high, comparable with those obtained by Tseng and Zhao [44] with yoghurt. Very different values were obtained by Marchiani et al. [38] for yoghurt and Marchiani et al. [45] for cheese, although this was due to the use of different extraction methods. 

### 3.4. Consumer Evaluation

Table 7 presents the results of the consumer evaluation performed for the tofu samples obtained with standard and innovative coagulants. The products obtained with magnesium chloride and citric acid showed high scores, although some tofu samples obtained with GPs were also characterised by high rank sum values. The most preferred products were those obtained with Barbera (red grape that produced a red-violet tofu) and Moscato (white aromatic grape). The tofu samples produced with Chardonnay and Pinot Noir were generally less favoured. The tofu samples made with GP concentrations of 5% were preferred, likely due to the more intense sensory characteristics.

## 4. Conclusions

Different types of tofu were prepared in this study using fresh and post-distillation GPs obtained using different grape varieties. Tofu samples coagulated with Moscato and Chardonnay pomaces were very similar, with a “woody” colour and semi-solid texture. These tofu samples were characterised by a high quantity of sugars, similar to those obtained with fresh pomace. The tofu produced with the pomace of Pinot Noir was found to be the most consistent and dry. Finally, the tofu produced with Barbera pomace created some difficulties during production due to the formation of very fine curds, and consequently its soft and almost spreadable consistency. These two tofu samples were characterised by higher total polyphenol contents and radical scavenging activity levels. All of the obtained tofu samples were naturally coloured, and this characteristic is important from a commercial point of view. Generally, there were no differences between tofu samples according to the distillation process, while there were significant differences due to grape variety. 

From the point of view of sensory characteristics, the tofu made using Barbera pomace had the greatest acceptability, although these results need to be confirmed by new and specific tests carried out with a greater number of consumers.

The tofu obtained with pomace is achievable on a technological level, as pomace naturally contains salts and acids; furthermore, since GPs are rich in polyphenolic compounds, they give tofu a functional value.

An ideal coagulant should be economical (i.e., affordable in most developing countries and especially in the least developed countries), convenient, have an efficient extraction rate, have possible use as an antioxidant, and have other functional properties, in addition to being highly nutritional and environmentally friendly. GPs have all of these characteristics, and the reuse of such pomaces in a new production process is valuable in order not to waste them. Their utilisation can also decrease the costs of winemaking. 

## Figures and Tables

**Table 1:** Results (X—mean; σ—standard deviation) of the compositional analysis of the tofu samples obtained with the traditional and innovative coagulants at two concentrations (2.5% and 5%) (BA—Barbera; CH—Chardonnay; PN—Pinot Noir; MO—Moscato; MAG—magnesium chloride; CIT—citric acid; PR—pre-distillation; PC—post-distillation continuous; PD—post-distillation discontinuous).

Coagulant		Dry Extract (%)		Ash (%)		Protein (%)		Carbohydrates (%)		Fats (%)		pH		a_w_	
Type	%	X	σ	X	σ	X	σ	X	σ	X	σ	X	σ	X	σ
MAG	-	14.91	0.24	0.93	0.09	8.94	0.86	2.41	0.25	2.63	0.26	5.98	0.01	1.01	0.01
CIT	-	14.90	0.31	0.47	0.09	8.44	0.84	2.80	0.26	3.19	0.32	5.18	0.05	1.01	0.00
BA	2.5	16.63	0.15	0.67	0.07	6.56	0.66	6.44	0.30	2.96	0.30	4.60	0.09	1.01	0.01
	5	18.61	0.18	0.95	0.10	5.25	0.53	9.99	0.32	2.42	0.24	4.20	0.11	1.00	0.01
MO	2.5	19.10	0.21	0.62	0.06	7.06	0.71	8.76	0.29	2.66	0.27	4.95	0.06	1.01	0.01
	5	19.86	0.23	0.68	0.07	5.44	0.54	11.71	0.28	2.03	0.20	4.50	0.09	1.00	0.01
CH	2.5	18.97	0.14	0.64	0.06	6.69	0.67	8.62	0.26	3.02	0.30	4.99	0.07	1.00	0.01
	5	21.79	0.25	0.82	0.08	6.13	0.61	11.99	0.31	2.85	0.29	4.52	0.10	1.01	0.01
PN	2.5	21.12	0.30	0.62	0.06	6.75	0.68	10.08	0.30	3.67	0.37	5.47	0.07	1.01	0.01
	5	23.10	0.25	0.69	0.07	6.69	0.67	12.45	0.24	3.27	0.33	5.02	0.11	1.01	0.01
BA_PR	2.5	21.73	0.28	0.74	0.07	7.94	0.79	9.10	0.22	3.95	0.40	4.67	0.02	1.01	0.01
	5	21.70	0.19	0.94	0.09	5.13	0.51	13.57	0.23	2.06	0.21	4.24	0.01	1.01	0.01
BA_PC	2.5	17.00	0.18	0.59	0.06	5.94	0.59	8.56	0.25	1.91	0.19	4.50	0.01	1.01	0.01
	5	22.00	0.15	0.86	0.09	4.81	0.48	15.25	0.25	1.08	0.11	4.12	0.01	1.01	0.01
BA_PD	2.5	18.31	0.14	0.65	0.07	6.00	0.60	9.74	0.26	1.92	0.19	4.64	0.01	1.01	0.01
	5	21.13	0.19	0.75	0.08	5.88	0.59	13.30	0.27	1.20	0.12	4.23	0.00	1.01	0.00
MO_PR	2.5	21.42	0.16	0.68	0.07	7.00	0.70	11.50	0.25	2.24	0.22	5.03	0.15	1.00	0.00
	5	22.28	0.21	0.78	0.08	5.13	0.51	15.40	0.22	0.97	0.10	4.55	0.08	1.00	0.01
MO_PC	2.5	19.53	0.23	0.62	0.06	6.69	0.67	10.77	0.21	1.45	0.15	4.98	0.06	1.01	0.01
	5	19.05	0.25	0.76	0.08	4.56	0.46	12.33	0.20	1.40	0.14	4.59	0.11	1.00	0.00
MO_PD	2.5	17.53	0.26	0.57	0.06	5.94	0.59	9.14	0.31	1.88	0.19	4.96	0.16	0.99	0.02
	5	19.65	0.21	0.67	0.07	4.69	0.47	9.64	0.32	1.45	0.15	4.48	0.15	1.00	0.01
CH_PR	2.5	22.63	0.22	0.74	0.07	5.44	0.54	14.88	0.31	1.57	0.16	5.23	0.11	1.00	0.00
	5	23.75	0.21	0.91	0.09	6.50	0.65	14.26	0.30	2.08	0.21	4.73	0.06	1.00	0.01
CH_PC	2.5	19.47	0.23	0.74	0.07	8.25	0.83	7.65	0.29	2.83	0.28	5.28	0.01	1.01	0.01
	5	22.04	0.28	0.95	0.10	6.31	0.63	12.65	0.33	2.13	0.21	4.72	0.02	1.01	0.01
CH_PD	2.5	20.87	0.20	0.68	0.07	7.63	0.76	9.72	0.28	2.84	0.28	5.12	0.13	1.01	0.01
	5	22.19	0.19	0.87	0.09	6.44	0.64	12.68	0.26	2.20	0.22	4.69	0.10	1.00	0.00

**Table 2:** Values (X—mean; σ—standard deviation) of CIELab parameters (L*, a*, b*) of the tofu samples obtained with the traditional and innovative coagulants at two concentrations (2.5% and 5%) and results of the variance analysis with post hoc Duncan’s test calculated among all samples (BA—Barbera; CH—Chardonnay; PN—Pinot Noir; MO—Moscato; MAG—magnesium chloride; CIT—citric acid; PR—pre-distillation; PC—post-distillation continuous; PD—post-distillation discontinuous).

	L *			a *			b *	
	X	σ		X	σ		X	σ
BA_PC 5%	20.48	0.07 a	MAG	0.27	0.26 a	BA_PR 2.5%	−1.51	0.79 a
BA_PR 5%	22.01	1.46 b	CIT	0.53	0.26 b	BA_PR 5%	−1.11	0.73 b
BA_PD 5%	22.53	0.36 b	PN 2.5%	6.47	0.21 c	BA_PC 2.5%	−0.49	0.30 c
BA_PC 2.5%	23.72	0.65 c	MO 2.5%	6.63	0.25 c	BA 5%	−0.45	0.33 c
BA_PR 2.5%	24.58	0.31 d	MO_PC 2.5%	6.81	0.22 d	BA 2.5%	0.09	0.44 d
BA_PD 2.5%	25.73	0.76 e	CH 2.5%	7.2	0.10 e	BA_PC 5%	0.26	0.25 d
BA 5%	25.76	0.56 e	BA_PR 2.5%	7.39	0.38 f	BA_PD 2.5%	1.22	0.25 e
BA 2.5%	31.2	0.44 f	PN 5%	7.52	0.25 f	BA_PD 5%	1.56	0.14 f
CH_PD 5%	33.55	0.39 g	MO_PC 5%	7.54	0.34 f	PN 2.5%	10.21	0.30 g
CH_PC 5%	35.15	0.60 h	CH_PC 2.5%	7.92	0.26 g	PN 5%	10.95	0.42 h
CH_PC 2.5%	36.6	2.66 i	MO_PD 2.5%	7.92	0.38 g	MO_PD 2.5%	13.46	0.47 i
CH_PR 5%	37.02	1.29 il	CH_PR 2.5%	7.94	0.47 g	MO_PD 5%	14.86	0.70 l
PN 5%	37.41	0.46 lm	MO_PR 2.5%	8.11	0.25 h	MO_PC 2.5%	14.95	0.34 lm
CH_PD 2.5%	37.73	0.45 mn	MO 5%	8.2	0.18 hi	CH_PC 2.5%	15.1	0.25 lm
MO_PD 5%	38.22	2.09 no	BA 2.5%	8.25	0.48 hil	CH_PR 2.5%	15.16	0.82 mn
PN 2.5%	38.5	0.77 op	CH 5%	8.31	0.22 ilm	CH_PD 2.5%	15.42	0.21 no
MO_PR 5%	38.97	1.29 pq	BA_PR 5%	8.42	0.24 lmn	CIT	15.52	0.33 o
CH_PR 2.5%	39.47	3.27 qr	CH_PD 2.5%	8.46	0.09 mn	MAG	15.58	0.44 op
MO_PC 5%	39.71	1.14 rs	CH_PC 5%	8.52	0.28 no	CH_PC 5%	15.84	0.85 p
MO_PD 2.5%	40.23	2.04 s	MO_PD 5%	8.67	0.52 o	CH_PR 5%	16.16	0.64 q
MO_PR 2.5%	41.09	1.80 t	CH_PR 5%	8.96	0.23 p	MO_PC 5%	16.43	0.69 q
MO_PC 2.5%	41.42	1.31 t	BA_PC 2.5%	8.97	0.37 p	CH_PD 5%	16.85	0.33 r
CH 5%	42.73	0.47 u	MO_PR 5%	9.21	0.22 q	MO_PR 2.5%	16.94	0.32 r
MO 5%	44.39	0.61 v	CH_PD 5%	9.55	0.16 r	CH 2.5%	17.62	0.36 s
CH 2.5%	44.96	0.69 v	BA_PD 2.5%	9.77	0.08 s	MO_PR 5%	18.64	0.25 t
MO 2.5%	46.75	0.70 w	BA 5%	10.54	0.25 t	MO 2.5%	18.93	0.53 u
MAG	81.55	0.78 x	BA_PC 5%	10.57	0.13 t	CH 5%	19.81	0.49 v
CIT	82	0.53 x	BA_PD 5%	10.92	0.14 u	MO 5%	21.87	0.54 w
Significance	***			***			***	

Note: *** *p* < 0.001; a–x, means in the same column followed by a different letter are significantly different (*p <* 0.05) according to Duncan’s test.

**Table 3:** Values of ΔE calculated among the tofu samples obtained with the traditional and innovative coagulants at two concentrations (2.5% and 5%) (BA—Barbera; CH—Chardonnay; PN—Pinot Noir; MO—Moscato; MAG—magnesium chloride; CIT—citric acid; PR—pre-distillation; PC—post-distillation continuous; PD—post-distillation discontinuous).

	MAG	CIT	BA 2.5	BA 5	CH 2.5	CH 5	MO 2.5	MO 5	PN 2.5	PN 5	BA_PR 2.5	BA_PR 5	BA_PC 2.5	BA_PC 5	BA_PD 2.5	BA_PD 5	CH_PR 2.5	CH_PR 5	CH_PC 2.5	CH_PC 5	CH_PD 2.5	CH_PD 5	MO_PR 2.5	MO_PR 5	MO_PC 2.5	MO_PC 5	MO_PD 2.5
MAG																											
CIT	0.5																										
BA2.5	53.3	53.6																									
BA5	58.9	59.3	5.9																								
CH2.5	37.3	37.7	22.3	26.6																							
CH5	39.9	40.3	22.8	26.5	3.3																						
MO2.5	35.5	35.9	24.5	28.8	2.3	4.4																					
MO5	38.5	38.9	25.5	29.2	4.4	2.6	4.1																				
PN2.5	43.8	44.2	12.6	17.1	9.9	10.7	12.0	13.2																			
PN5	45.0	45.4	12.5	16.6	10.1	10.4	12.3	13.0	1.7																		
BA_PR2.5	59.9	60.3	6.9	3.5	28.0	28.0	30.2	30.7	18.2	17.9																	
BA_PR5	62.4	62.7	9.3	4.4	29.6	29.4	31.9	32.1	20.1	19.6	2.8																
BA_PC2.5	60.6	61.0	7.5	2.6	28.0	27.8	30.2	30.5	18.4	17.9	2.1	1.9															
BA_PC5	63.8	64.2	11.0	5.3	30.2	29.7	32.5	32.3	21.0	20.3	5.5	3.0	3.7														
BA_PD2.5	58.4	58.8	5.8	1.8	25.4	25.2	27.7	27.9	16.0	15.4	3.8	4.6	2.8	5.4													
BA_PD5	61.6	62.0	9.2	3.8	27.8	27.4	30.1	30.0	18.7	17.9	5.1	3.7	3.1	2.5	3.4												
CH_PR2.5	42.8	43.2	17.2	20.9	6.1	5.7	8.3	8.3	5.3	4.7	22.4	23.9	22.2	24.3	19.7	21.9											
CH_PR5	45.4	45.8	17.1	20.1	8.3	6.8	10.4	9.4	6.6	5.4	21.7	22.9	21.3	23.0	18.7	20.7	2.8										
CH_PC2.5	45.6	46.0	16.0	19.1	8.8	7.7	10.9	10.3	5.4	4.2	20.5	21.8	20.2	22.1	17.7	19.8	2.9	1.6									
CH_PC5	47.1	47.5	16.2	18.9	10.1	8.6	12.2	11.0	6.9	5.5	20.3	21.4	19.9	21.5	17.4	19.2	4.4	1.9	1.7								
CH_PD2.5	44.6	45.0	16.7	20.0	7.7	6.7	9.8	9.3	5.6	4.6	21.5	22.8	21.2	23.1	18.6	20.7	1.8	1.1	1.3	2.6							
CH_PD5	48.9	49.3	17.0	19.0	11.7	9.7	13.7	12.0	8.8	7.3	20.6	21.4	19.9	21.2	17.5	18.9	6.4	3.6	3.9	2.2	4.5						
MO_PR2.5	41.2	41.6	19.5	23.3	4.0	3.3	6.2	5.9	7.4	7.1	24.8	26.3	24.6	26.6	22.0	24.3	2.4	4.2	4.9	6.1	3.7	7.7					
MO_PR5	43.6	44.0	20.1	23.3	6.4	4.0	8.2	6.4	8.9	8.0	24.8	26.0	24.5	26.1	21.9	23.8	3.7	3.2	4.5	4.8	3.5	5.7	2.9				
MO_PC2.5	40.7	41.1	18.1	22.3	4.5	5.3	6.7	7.7	5.6	5.7	23.6	25.2	23.6	25.9	21.1	23.5	2.3	5.1	4.9	6.6	4.1	8.5	2.4	5.0			
MO_PC5	42.5	42.9	18.4	22.1	5.4	4.6	7.5	7.2	6.4	5.9	23.5	24.9	23.3	25.3	20.8	23.0	1.4	3.1	3.4	4.7	2.4	6.5	1.6	2.9	2.4		
MO_PD2.5	42.1	42.5	16.1	20.2	6.3	6.8	8.6	9.4	4.0	3.8	21.7	23.3	21.6	23.9	19.1	21.5	1.9	4.3	4.0	5.6	3.2	7.7	3.6	5.5	2.2	3.0	
MO_PD5	44.1	44.5	16.4	19.8	7.4	6.7	9.7	9.3	5.2	4.2	21.3	22.8	21.1	23.1	18.5	20.7	1.5	1.8	1.8	3.2	0.8	5.2	3.6	3.9	3.7	2.4	2.6

**Table 4:** Results of the texture analysis (X—mean; σ—standard deviation) for the tofu samples obtained with the traditional and innovative coagulants at two concentrations (2.5% and 5%) and results of variance analysis with post hoc Duncan’s test (BA—Barbera; CH—Chardonnay; PN—Pinot Noir; MO—Moscato; MAG—magnesium chloride; CIT—citric acid; PR—pre-distillation; PC—post-distillation continuous; PD—post-distillation discontinuous).

	Hardness			Cohesiveness			Adhesiveness			Gumminess			Chewiness			Springiness			Resilience	
	X	σ		X	σ		X	σ		X	σ		X	σ		X	σ		X	σ
CIT	1.83	0.47 a	PN 5%	0.15	0.01 a	BA 5%	−3.11	1.45 a	BA_PD 2.5%	0.56	0.20 a	BA_PD 5%	1.61	0.24 a	PN 5%	2.34	0.14 a	BA 5%	0.03	0.00 a
MAG	2.32	0.57 ab	CH 5%	0.16	0.01 ab	BA_PC 5%	−1.91	1.29 b	CIT	0.60	0.26 abc	BA_PD 2.5%	1.82	0.87 a	MO_PR 5%	2.45	0.23 ab	BA_PC 5%	0.03	0.00 a
CH_PD 2.5%	2.40	0.18 abc	BA_PC 5%	0.16	0.02 ab	MO_PC 5%	−1.91	0.67 bc	BA_PD 5%	0.62	0.08 ab	MO 5%	2.00	0.21 a	CH 5%	2.46	0.19 ab	MO_PR 5%	0.03	0.00 a
BA_PD 2.5%	2.42	0.62 ab	MO_PR 5%	0.16	0.02 abc	CH_PC 5%	−1.88	0.67 bc	CH_PD 2.5%	0.64	0.05 abcd	MO_PR 5%	2.02	0.88 a	MO_PC 5%	2.62	0.43 ab	MO_PC 5%	0.03	0.00 a
BA_PR 2.5%	2.85	0.80 abcd	MO_PC 5%	0.16	0.02 abc	BA_PC 2.5%	−1.73	0.95 b	BA_PR 2.5%	0.69	0.21 abcd	BA_PC 5%	2.06	0.94 a	BA_PD 5%	2.63	0.36 abc	CH 5%	0.04	0.01 a
CH_PD 5%	3.02	0.46 bcde	BA_PD 5%	0.18	0.02 abcde	CH_PC 2.5%	−1.66	0.49 bcde	CH_PD 5%	0.69	0.11 abcde	CH_PR 5%	2.07	0.54 a	MO_PD 5%	2.72	0.34 ab	MO 5%	0.04	0.00 a
CH_PC 2.5%	3.17	1.20 bcdef	MO_PD 5%	0.18	0.03 abcd	MO_PD 5%	−1.63	1.08 bcd	BA_PC 5%	0.70	0.17 abcd	CH 5%	2.14	0.52 ab	MO 5%	2.83	0.16 abc	PN 5%	0.04	0.00 a
CH_PC 5%	3.33	0.43 bcdefg	MO 5%	0.19	0.01 abcde	BA_PD 5%	−1.48	0.80 bcde	MO 5%	0.71	0.04 abcdef	PN 5%	2.20	0.19 ab	CH_PR 5%	2.83	0.35 abc	BA_PR 5%	0.04	0.01 a
MO 2.5%	3.41	0.36 cdefg	PN 2.5%	0.19	0.01 abcde	BA 2.5%	−1.45	0.87 bcdef	CH_PC 2.5%	0.71	0.10 abcdef	MO_PC 2.5%	2.22	0.56 ab	BA_PC 5%	2.85	0.64 abc	BA_PC 2.5%	0.04	0.01 a
BA_PD 5%	3.43	0.62 bcdef	BA_PR 5%	0.20	0.04 abcde	MO_PD 2.5%	−1.40	0.47 bcdef	CH_PR 5%	0.72	0.12 abcdef	MO_PC 5%	2.24	0.62 ab	BA_PR 5%	2.89	0.52 abc	BA_PD 2.5%	0.04	0.00 a
BA 2.5%	3.48	1.87 defg	CH_PR 5%	0.20	0.04 abcde	MO_PC 2.5%	−1.33	0.34 bcdef	CH_PC 5%	0.74	0.13 abcdef	CH_PD 5%	2.32	0.45 ab	MO_PC 2.5%	2.9	0.16 abc	BA_PD 5%	0.04	0.01 a
CH_PR 5%	3.64	0.43 defgh	MO_PC 2.5%	0.20	0.02 abcde	MO_PR 2.5%	−1.31	0.25 bcdef	CH_PR 2.5%	0.76	0.12 abcdef	CH_PR 2.5%	2.38	0.70 ab	PN 2.5%	2.95	0.07 abc	CH_PR 2.5%	0.04	0.01 a
MO_PC 2.5%	3.77	0.67 efghi	BA_PC 2.5%	0.21	0.02 abcdef	BA_PD 2.5%	−1.27	0.85 bcdef	MO_PC 2.5%	0.76	0.17 abcdef	CH_PC 2.5%	2.39	0.59 ab	BA 2.5%	3.11	0.43 abcd	CH_PR 5%	0.04	0.01 a
CH 2.5%	3.78	0.49 efghi	CH_PR 2.5%	0.21	0.07 abcde	BA_PR 5%	−1.23	0.57 bcdef	BA 2.5%	0.81	0.34 abcdef	BA 2.5%	2.4	0.75 ab	BA_PD 2.5%	3.16	0.42 abcd	CH_PC 2.5%	0.04	0.01 a
MO 5%	3.78	0.32 efghi	BA_PD 2.5%	0.22	0.03 abcdef	BA_PR 2.5%	−1.22	0.57 bcdef	MO_PR 5%	0.81	0.30 abcdef	BA_PR 2.5%	2.41	0.87 ab	CH_PR 2.5%	3.19	0.95 abcd	CH_PC 5%	0.04	0.01 a
BA_PC 2.5%	3.89	0.83 efghi	CH_PC 5%	0.22	0.02 abcdef	CH 5%	−1.20	0.46 bcdef	BA_PC 2.5%	0.82	0.11 abcdef	CH_PC 5%	2.45	0.49 ab	BA_PC 2.5%	3.2	0.30 abcd	CH_PD 5%	0.04	0.00 a
CH_PR 2.5%	3.91	1.55 efghi	MO_PR 2.5%	0.22	0.04 abcdef	PN 5%	−1.15	0.20 bcdef	MAG	0.84	0.27 abcdef	BA_PR 5%	2.47	0.67 ab	CH_PC 5%	3.29	0.23 abcd	MO_PR 2.5%	0.04	0.01 a
MO_PR 2.5%	4.22	0.87 fghil	MO_PD 2.5%	0.22	0.03 abcde	MO_PR 5%	−1.14	0.28 bcdef	MO 2.5%	0.84	0.07 abcdef	PN 2.5%	2.61	0.48 ab	MO_PR 2.5%	3.29	0.57 abcd	MO_PC 2.5%	0.04	0.01 a
BA_PC 5%	4.24	0.69 efghi	CH_PD 5%	0.23	0.02 abcdef	CH_PD 5%	−1.12	0.32 bcdef	BA_PR 5%	0.85	0.14 abcdef	BA_PC 2.5%	2.61	0.16 ab	CH_PD 5%	3.33	0.19 abcd	MO_PD 5%	0.04	0.01 a
BA_PR 5%	4.46	0.91 ghilm	BA 2.5%	0.24	0.03 bcdef	CH_PR 2.5%	−1.11	0.60 bcdef	MO_PC 5%	0.85	0.18 abcdef	CH_PD 2.5%	2.73	0.47 ab	CH 2.5%	3.42	0.31 abcd	BA 2.5%	0.05	0.02 a
MO_PD 2.5%	4.53	0.61 hilm	BA_PR 2.5%	0.24	0.02 bcdef	CH_PR 5%	−1.08	0.21 bcdef	CH 5%	0.87	0.18 abcdef	MO_PD 5%	2.86	0.50 ab	BA_PR 2.5%	3.45	0.31 abcd	PN 2.5%	0.05	0.01 a
PN 2.5%	4.71	1.08 hilm	CH_PC 2.5%	0.24	0.06 abcde	CH_PD 2.5%	−1.08	0.22 bcdef	PN 2.5%	0.89	0.18 bcdef	MO_PR 2.5%	2.91	0.39 ab	CH_PC 2.5%	3.46	1.14 abcd	BA_PR 2.5%	0.05	0.00 a
BA 5%	4.73	1.29 hilm	MO 2.5%	0.25	0.02 cdef	CH 2.5%	−1.04	0.30 bcdef	MO_PR 2.5%	0.89	0.06 bcdef	CIT	3.13	2.34 ab	MO_PD 2.5%	3.64	1.16 abcd	CH_PD 2.5%	0.05	0.01 a
MO_PR 5%	4.83	1.24 ilm	CH 2.5%	0.26	0.11 def	MO 5%	−1.02	0.21 bcdef	PN 5%	0.94	0.03 cdef	MO 2.5%	3.14	0.32 ab	MO 2.5%	3.73	0.34 bcde	MO_PD 2.5%	0.05	0.01 a
MO_PC 5%	5.20	1.05 lmn	CH_PD 2.5%	0.27	0.03 efg	MO 2.5%	−0.95	0.22 cdef	CH 2.5%	0.97	0.43 def	CH 2.5%	3.35	1.60 ab	BA 5%	3.91	1.86 cdef	MO 2.5%	0.06	0.01 a
CH 5%	5.38	1.02 mno	BA 5%	0.30	0.15 fgh	PN 2.5%	−0.89	0.16 def	MO_PD 2.5%	0.98	0.21 ef	MO_PD 2.5%	3.65	1.74 ab	CH_PD 2.5%	4.23	0.50 adef	MAG	0.17	0.15 ab
MO_PD 5%	5.78	0.81 no	CIT	0.34	0.11 gh	CIT	−0.70	1.18 ef	MO_PD 5%	1.05	0.10 f	MAG	4.45	2.77 bc	CIT	4.76	1.95 ef	CIT	0.22	0.24 ab
PN 5%	6.30	0.29 o	MAG	0.37	0.10 h	MAG	−0.50	0.53 f	BA 5%	1.34	0.71 g	BA 5%	5.91	5.61 c	MAG	4.93	1.64 f	CH 2.5%	0.24	0.58 b
Significance	***			***			***			***			***			***			***	

Note: *** *p* < 0.001; a–x, means in the same column followed by different letters are significantly different (*p <* 0.05) according to Duncan’s test.

**Table 5:** Values (X—mean; σ—standard deviation) of radical scavenging activity (RSA; μmol TE/g) and polyphenolic content (TPC; mg GAE/g) of GPs used as coagulants and results of variance analysis with post hoc Duncan’s test (BA—Barbera; CH—Chardonnay; PN—Pinot Noir; MO—Moscato; MAG—magnesium chloride; CIT—citric acid; PR—pre-distillation; PC—post-distillation continuous; PD—post-distillation discontinuous).

	RSA			TPC	
	X	σ		X	σ
CH_PD	59.74	3.01 a	CH_PD	11.65	0.13 a
CH_PR	72.98	8.41 b	CH_PR	14.18	0.34 b
CH_PC	88.43	4.00 c	MO_PD	16.81	2.60 c
BA_PR	116.38	12.64 d	CH_PC	18.31	0.95 cd
MO_PR	120.79	6.56 d	CH	18.36	0.64 cd
BA_PD	131.47	3.67 e	BA_PR	19.68	0.32 de
MO_PD	166.53	5.23 f	MO	20.87	1.63 e
CH	171.65	9.55 f	MO_PR	22.87	1.39 f
MO	181.94	4.90 g	BA_PC	23.94	2.94 fg
BA_PC	190.15	3.76 g	BA_PD	24.83	1.82 g
MO_PC	206.89	9.23 h	MO_PC	24.95	1.28 g
PN	266.67	6.37 i	PN	38.85	1.40 h
BA	300.88	10.24 l	BA	38.88	0.68 h
Significance	***			***	

Note: *** *p* < 0.001; a–l, means in the same column followed by different letters are significantly different (*p <* 0.05) according to Duncan’s test.

**Table 6:** Values (X—mean; σ—standard deviation) of radical scavenging activity (RSA; μmol TE/g) and polyphenolic content (TPC; mg GAE/g) of tofu obtained with the traditional and innovative coagulants at two concentrations (2.5% and 5%) and results of variance analysis with post hoc Duncan’s test (BA—Barbera; CH—Chardonnay; PN—Pinot Noir; MO—Moscato; MAG—magnesium chloride; CIT—citric acid; PR—pre-distillation; PC—post-distillation continuous; PD—post-distillation discontinuous).

	RSA			TPC	
	X	σ		X	σ
MAG	1.98	0.05 a	MAG	1.43	0.07 a
CIT	2.22	0.06 a	CIT	1.59	0.10 a
CH_PD 2.5%	9.26	0.14 b	CH_PD 2.5%	2.52	0.12 b
MO 2.5%	9.62	0.86 bc	MO 2.5%	2.70	0.24 bc
MO 5%	13.3	0.43 bcd	MO 5%	2.82	0.08 bc
CH_PR 2.5%	13.84	3.88 bcde	CH_PC 2.5%	2.99	0.11 bcd
MO_PR 2.5%	14.31	3.44 cde	MO_PR 2.5%	3.08	0.60 bcd
CH 2.5%	14.66	0.29 cde	CH_PR 2.5%	3.10	0.41 bcd
BA 2.5%	15.27	0.39 de	CH_PD 5%	3.17	0.18 bcd
CH_PD 5%	15.29	0.63 de	CH 2.5%	3.59	0.12 cd
BA_PR 2.5%	16.97	2.31 de	BA_PR 2.5%	3.66	0.51 cd
CH_PC 2.5%	18.11	5.47 de	CH_PR 5%	3.74	0.14 cde
CH_PR 5%	18.97	2.45 ef	BA 2.5%	3.90	0.12 def
BA_PD 2.5%	23.07	1.08 fg	CH_PC 5%	4.66	0.48 efg
CH_PC 5%	23.34	4.23 fg	MO_PC 2.5%	4.80	0.80 fg
MO_PC 2.5%	25.20	3.78 g	MO_PR 5%	4.87	0.44 fgh
MO_PR 5%	25.43	2.37 g	MO_PD 2.5%	5.53	2.06 ghi
BA_PR 5%	26.73	0.50 g	BA_PR 5%	5.65	0.38 ghi
BA_PC 2.5%	31.26	5.51 h	BA_PC 2.5%	5.83	0.64 hi
BA 5%	32.80	0.46 hi	CH 5%	5.84	0.79 hi
CH 5%	34.20	3.59 hil	BA 5%	5.88	0.22 hi
MO_PD 2.5%	34.61	12.87 hil	MO_PD 5%	6.09	1.05 il
PN 2.5%	37.67	3.76 ilm	PN 2.5%	6.33	0.59 il
BA_PD 5%	38.50	1.52 lm	BA_PD 5%	6.44	0.09 il
MO_PD 5%	42.39	6.90 m	BA_PD 2.5%	6.94	2.63 lm
MO_PC 5%	47.95	8.91 n	MO_PC 5%	7.61	1.74 m
BA_PC 5%	50.43	5.81 n	BA_PC 5%	8.79	0.84 n
PN 5%	80.51	2.94 o	PN 5%	10.34	0.72 o
Significance	***			***	

Note: *** *p* < 0.001; a–o, means in the same column followed by different letters are significantly different (*p <* 0.05) according to Duncan’s test.

**Table 7:** Results (rank sums and results of Kruskal–Wallis test) of the consumer evaluation of the tofu samples obtained with the traditional and innovative coagulants at two concentrations (2.5% and 5%) (BA—Barbera; CH—Chardonnay; PN—Pinot Noir; MO—Moscato; MAG—magnesium chloride; CIT—citric acid; PR—pre-distillation; PC—post-distillation continuous; PD—post-distillation discontinuous).

	Overall Liking	
CH 2.5%	7832	a
MO_PR 5%	8324	ab
PN 2.5%	8504	abc
CH_PC 2.5%	8521	abc
BA 2.5%	8753	abc
BA_PR 2.5%	8973	abc
CH_PR 2.5%	9329	abcd
CH_PD 2.5%	9577	abcd
MO_PC 2.5%	10,063	abcde
PN 5%	10,513	abcde
BA_PC 5%	10,942	abcde
CH_PR 5%	11,033	abcde
MO 5%	11,065	abcde
BA_PD 5%	11,481	abcde
MO_PD 2.5%	11,487	abcde
MO_PR 2.5%	11,578	abcde
CH_PD 5%	11,616	abcde
CIT	11,708	abcde
CH_PC 5%	11,758	abcde
MO_PC 5%	11,810	abcde
BA_PC 2.5%	11,862	bcde
BA_PD 2.5%	11,997	bcde
MO 2.5%	12,133	cde
BA_PR 5%	12,241	bcde
CH 5%	12,871	cde
MO_PD 5%	13,211	de
BA 5%	14,250	e
MAG	14,293	e

Note: *** *p* < 0.001; a–e, rank sum followed by different letters are significantly different (*p <* 0.05).
